# Comparing UAV-Based Technologies and RGB-D Reconstruction Methods for Plant Height and Biomass Monitoring on Grass Ley

**DOI:** 10.3390/s19030535

**Published:** 2019-01-28

**Authors:** Victor P. Rueda-Ayala, José M. Peña, Mats Höglind, José M. Bengochea-Guevara, Dionisio Andújar

**Affiliations:** 1 Department of Grassland and Livestock, Norwegian Institute of Bioeconomy Research, NIBIO Særheim, Postvegen 213, 4353 Klepp Stasjon, Norway; patovicnsf@gmail.com (V.P.R.-A.); mats.hoglind@nibio.no (M.H.); 2 Institute of Agricultural Sciences, Consejo Superior Investigaciones Científicas (CSIC), Serrano 115b, 28006 Madrid, Spain; jmpena@ica.csic.es; 3 Centre for Automation and Robotics, Consejo Superior Investigaciones Científicas (CSIC), Ctra. de Campo Real km 0.200 La Poveda, 28500 Arganda del Rey (Madrid), Spain; jose.bengochea@car.upm-csic.es

**Keywords:** 3D crop modeling, remote sensing, on-ground sensing, depth images, parameter acquisition

## Abstract

Pastures are botanically diverse and difficult to characterize. Digital modeling of pasture biomass and quality by non-destructive methods can provide highly valuable support for decision-making. This study aimed to evaluate aerial and on-ground methods to characterize grass ley fields, estimating plant height, biomass and volume, using digital grass models. Two fields were sampled, one timothy-dominant and the other ryegrass-dominant. Both sensing systems allowed estimation of biomass, volume and plant height, which were compared with ground truth, also taking into consideration basic economical aspects. To obtain ground-truth data for validation, 10 plots of 1 m^2^ were manually and destructively sampled on each field. The studied systems differed in data resolution, thus in estimation capability. There was a reasonably good agreement between the UAV-based, the RGB-D-based estimates and the manual height measurements on both fields. RGB-D-based estimation correlated well with ground truth of plant height ($R^{2}>0.80$) for both fields, and with dry biomass ($R^{2}=0.88$), only for the timothy field. RGB-D-based estimation of plant volume for ryegrass showed a high agreement ($R^{2}=0.87$). The UAV-based system showed a weaker estimation capability for plant height and dry biomass ($R^{2}<0.6$). UAV-systems are more affordable, easier to operate and can cover a larger surface. On-ground techniques with RGB-D cameras can produce highly detailed models, but with more variable results than UAV-based models. On-ground RGB-D data can be effectively analysed with open source software, which is a cost reduction advantage, compared with aerial image analysis. Since the resolution for agricultural operations does not need fine identification the end-details of the grass plants, the use of aerial platforms could result a better option in grasslands.

## 1. Introduction

Pastures are botanically diverse and difficult to characterize, due to their complex species composition. A good characterization of a forage crop parameters is crucial for successful grassland management [1]. Technological advancement and current sensing technologies are powerful tools for elaborating accurate plant architecture models for phenotyping [2,3]. Digital modeling can be used to detect environmental stress problems, diseases, or the necessity of applying agricultural operations, at the right location and timing. Sensor data could be acquired throughout the whole plant life cycle and be available for model development and validation [4]. Spatial and temporal crop parameter information, for instance biomass and nitrogen content [5], add up value to vegetation models [6], strengthening decision-support systems for site-specific agronomic applications. Because perennial crops have a great biomass building potential, continuous supervision of crop development parameters along their life cycle is recommended [7]. Continuous supervision by means of spatial models could improve decision-making for forage grass production. Moreover, spatial models facilitate estimation of above-ground biomass, canopy height or plant cover in a non-destructive manner, which allow better programming of specific tasks, such as cutting time, fertilization and grasslands renewal [8].

Spatial vegetation models are based on data from digital imaging, spectrometry, fluorescence, thermal or distance measurements, which relate to some plant traits. Spectral reflectance of plant leaves, ranging from ultraviolet (UV), through visible light and near-infrared (NIR) and infrared (IR) wavelengths have been found to be particularly important for calculation of various vegetation indices [9,10]. Vegetation indices often correlate with leaf area index (LAI), biomass or dry matter yield [11]. Biomass estimation using the normalized difference vegetation index (NDVI) has given good results on annual pastures under grazing, although, poor data quality caused large estimation discrepancies on grazed or partially grazed paddoks [12]. Ground-based and aerial visible imaging data acquired at a specific time and the use of algorithms able to segment the RGB spectrum have been proposed for quick and simple description of plant growing dynamics [5,13,14]. However, data assessment from RGB images is limited in some aspects, such as leaf overlapping, that can make important parts of the plant difficult to detect, especially in grass mixtures. Distance sensors can measure distances by different principles (e.g., time-of-flight) and enable estimation of plant height or derive biomass weight by indirect relationships with height [15,16]. Distance sensors, which are normally divided into ultrasonic devices and LiDAR (Light Detection and Ranging), have been widely applied in modern agriculture operations [17,18,19,20]. Because they are easy to handle, these sensors can be used to assess big field areas in short time.

The use of 3D technologies from on-ground or aerial platforms open new scenarios for plant modeling. Characterization of plants with the aid of 3D models is available for use in breeding programs and agricultural decision making [8,21]. Various processes are available for capturing the three dimensions, height, width and depth, as 3D point clouds with X-Y-Z coordinates. The most explored, fastest and accurate 3D sensing system is LiDAR combined with sequential displacement of the sensor to acquire the Z coordinate [22]. A drawback of this system is the requirement of calibration and displacement across the sampling space, which increases the associated costs, as the resolution increases [23]. Fortunately, RGB-Depth (RGB-D) cameras and image processing based on Time-of-Flight can compensate those drawbacks by combining depth information with the color scene in a single shoot [17]. RGB-D cameras have been used for several agricultural research and application purposes. The most common is Microsoft Kinect^®^ v2, which allows reconstruction of 3D models, associated with color information. Microsoft launched the fist version of this development in 2010 with the Kinect, and since then, several other devices have appeared in the market: RGB-D cameras, such as the Intel RealSense, Primesense Carmine, Google Tango, or Occiptial’s Structure Sensor. These sensors are available at low price, and can capture pixel color and depth images at adequate resolution and at a high rate. Due to the similar output produced by those, the reconstruction method can be easily replicated. This methodology has been successfully applied on many crops, except on grasslands. Wang and Li [24] calculated onion volume with a high accuracy, compared to real measurements. Foliar density of threes was estimated for autonomous spraying [25]. Andújar et al. [17] used a dual methodology separating crops and weeds from soil in maize crops under field conditions. The latter methodology included height selection and RGB segmentation, using a unique model for plant discrimination. Combination of various frames allows reconstruction of big crop surface areas [21,26]. Live use of RGB-D on outdoors scenarios is possible with the current version of Kinect^®^ v2.

UAV’s can cover large areas and operate independently of soil conditions [27], which allows more flexibility than ground-based systems, at reduced operational time and costs. Photogrammetry on aerial imagery has shown a high functionality in different studies. High spatial resolution images can be obtained when flying at low altitudes, with large overlapping between images. The data can be processed through Structure-from-Motion reconstruction for building the 3D model. This method has been tested in olive trees to calculate canopy area, tree height and crown volume by generation of digital surface models and OBIA algorithm [28]. Hyperspectral aerial imagery can be used to calculate plant height and values related with dry biomass [29]. Nevertheless, this technology is rapidly improving for application in complex grassland scenarios [30].

Current challenges in both, agricultural research and production rely on sensing devices and technological advancement directed to improve crop quality and increment yield levels. As for other crop producers, forage farmers can immensely benefit from advanced technological support of digital grass modeling, to enhance forage productivity. Digital models could objectively deal with the complexity of grass-mixtures, and assist in the optimization of inputs, leading to better distribution or reduction of fertilizers, pesticides or seeds, e.g., by site-specific fertilization and renewing grass mixtures. In addition, some grassland farming activities in the field depend on biomass estimation to evaluate productivity, normally done via destructive methods (i.e., in this study referring to cutting numerous grass samples). Therefore, this study was carried out with the aim of evaluating aerial and on-ground methods to characterize grass ley fields, composed of different species mixtures. Specifically, it was attempted to objectively estimate plant height, biomass and volume, using digital grass models, and avoiding the unnecessary destruction of the swards.

## 2. Materials and Methods

### 2.1. Experiments and Modeling Systems

**Figure 1:**
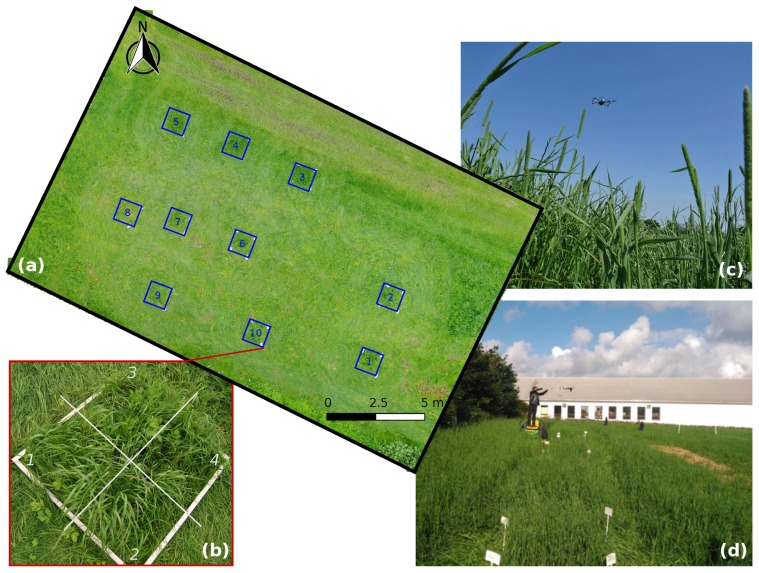
Field test conducted at NIBIO Særheim, orthophoto of the ryegrass-dominant field (**a**) with 10 sampling plots and a zoomed in 1 m^2^ sampling plot subdivided in four quadrants (**b**); UAV sampling system (**c**) and RGB-D sampling system (**d**).

Two digital characterization systems, a on-ground and a UAV-based system, were used to map pasture architecture on two fields located at NIBIO Særheim research station (Klepp Stasjon, Norway, lat. 58.76 N, long. 5.65 E). The site is characterized by a cold maritime climate with cool summers and cold winters. The precipitation is about 1180 mm annually, especially in autumn and spring. The on-ground system used a RGB-D camera, while the UAV-based sytem used a RGB camera with geo-positioning (geo-tagging) for data acquisition. These systems were tested and compared previously on a small area [31], at the same location. Two fields, each of 0.5 ha in size, were mapped. To obtain ground-truth data for validation, 10 plots of 1 m^2^ were sampled on each field (Figure 1a). Each plot was subdivided in four quadrants for measuring the variability within the 1 m^2^ area (Figure 1c). Field 1 (ryegrass dominant) was composed of 80% perennial ryegrass (*Lolium perenne* L.), 5% annual ryegrass *Lolium multiflorum* L. and 15% white clover (*Trifolium repens* L.). Field 2 (timothy dominant) was composed of 85% timothy (*Phleum pratense* L.), 10% perennial ryegrass, and 5% annual ryegrass. Both fields were established in 2015. From 2016, they were fertilized annually with 10, 8 and 6 tons of liquid manure at early spring (March–April), after first cut (June–July) and after second cut (August–September), respectively, corresponding to about 260 kg ha^−1^ year^−1^. Field assessments were conducted during July–August 2017 when the swards were fully developed, at anthesis stage.

For the on-ground system the RGB-D Microsoft Kinect^®^ v2 (Microsoft, Redmond, WA, USA) was used, as described by Andújar et al. [17]. Kinect V2 is the most widely used among RGB-D sensors. Although the device is no longer supported by Microsoft, its capabilities are similar to any other option in the market. In addition, readings sensors of this type show a common output, and the processing methodology is similar. The device is equipped with a standard RGB camera of 1080p, a depth camera, an infrared camera and an array of microphones. The RGB camera has a resolution of 1920×1080, which can adapt automatically the exposure time of the RBG to obtain brighter images at limiting light conditions. The IR camera can take a clear view into the darkness with a resolution of 512×424 pixels. The opening field of view (FOV) is different for every camera. The IR camera has a FOV of 70 degrees horizontally and depth perception is limited to 60 degrees vertically. The range of depth that can be measured with this camera goes from 0.5 to 4.5 m of distance from the sensor, although in outdoors conditions, the maximum range decreases. Studies conducted outdoors under different daytime illumination conditions showed valid depth measurements up to 1.9 m during sunny days, while the distance increases up to 2.8 m under the diffuse illumination of an overcast day [17]. The req uired overlap to fuse the acquired images and create the models is reached by a frame rate than can be set up to 30 fps during data acquisition. The distance is calculated for every pixel in the scene by the method of Time-of-Flight method by phase detection, i.e., the distance is calculated based on the time that a pulse of light takes to travel from the light source to the impacted plant and back to the sensor.

**Figure 2:**
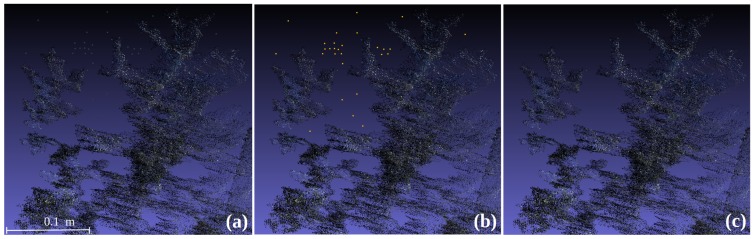
Section of the 3D reconstruction: before filtering (**a**); removed points are marked in fluorescent (**b**) and after filtering (**c**).

An Intel laptop computer with Windows 8 supported by Kinect SDK (software development kid) was used for data collection. The SDK helps acquiring data by classes, functions and structures, providing the necessary drivers for the sensor, and some sample functions that were implemented for the measurements combined with some OpenCV (The Open Source Computer Vision https://www.opencv.org/). The sensor was hand held pointing out the field samples from top view. The developed method for point cloud generation and reconstruction of large regions, using the fusion of different overlapped depth images was based on a previous development [32]. Storing information only on the voxels closest to the detected object and accessing to the stored information by using a hash table. Following that, for every new input depth image and knowing camera position, the ray-casting technique [33] was applied to project a ray from the camera focus for each pixel of the input depth image to determine the voxels in the 3D world crossing each ray. Then, the voxels related to the depth information are determined. Next step was conducted with a variant of the iterative closest point (ICP) algorithm, which provides a point cloud as output. Thus, the modified algorithm creates a point cloud by detecting the overlapping areas in sequential frames by assessing the relative position of the Kinect sensor for each frame to create a 3D model and removing outliers from the mesh [26]. Outliers could appear isolated in the point cloud. A point was considered an outlier if the average distance to its 64 nearest neighbours is greater than the standard deviation of the distance to the neighbours of all the points (Figure 2). The time to complete the acquisition was lower than 2 s from the top view. The system was supplied with electric power by a field vehicle that allows field measurement and support every device used during the acquisition process.

**Figure 3:**
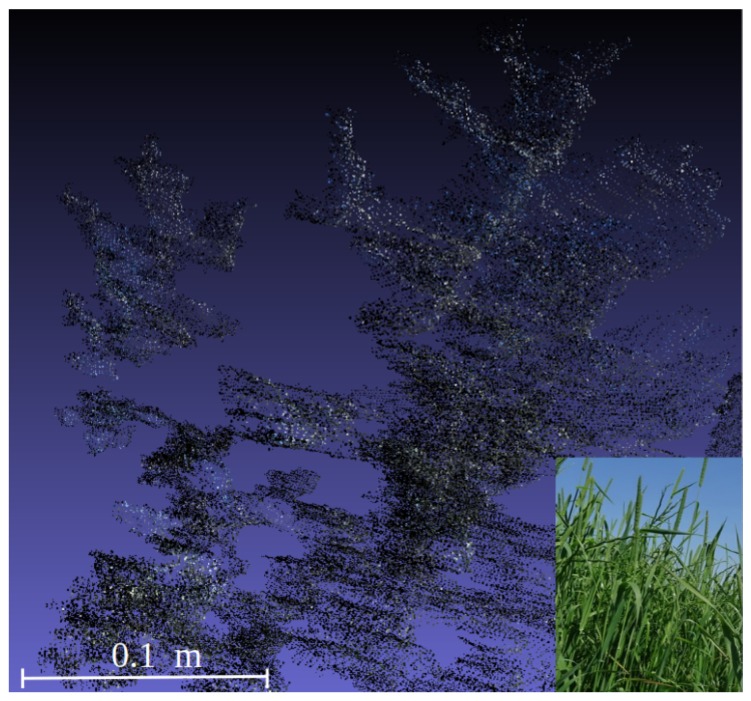
Point clouds created by RGB-D (Microsoft Kinect^®^ v2) system.

**Figure 4:**
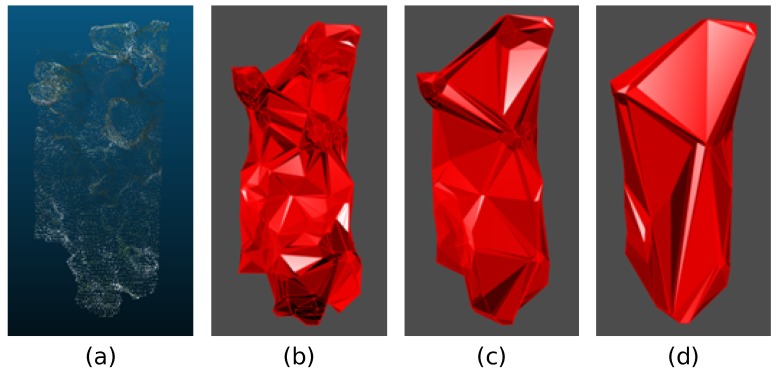
Alpha shapes for the same point cloud using alpha = 0.1 (**a**) and (**b**), alpha = 0.2 (**c**) and alpha = 0.4 (**d**).

One 3D model was build on the sward before harvesting and one inmmediately after. Once those 3D representations of the sampled plots were available (Figure 3), plant height and volumes could be estimated. For this purpose, both models were overlayed and plant height was estimated by difference between the two models, using cloudcompare. Firstly, an alpha shape [34] or volume that enveloped the set of 3D points was obtained. The alpha parameter specifies how tight the body fits the points. To address this issue, the R package alphashape3d [35] was employed. Figure 4 shows different alpha shapes according to the alpha value selected for the same point cloud. Higher values showed very loose shapes, whereas lower values generated tight bodies. The volume was estimated by applying the same function library that allows calculation of the alpha shapes.

The aerial system consisted of a DJI Mavic Pro quadcopter, combining a 4K digital camera and location information, was used for aerial imaging. The camera mounted on the UAV had a 28 mm lens with a Field of Fiew (FOV) of 78.8 degrees and a resolution of 4000 × 3000, capable of shooting 12.35 megapixel photos; the camera was 3-axis stabilized by its drone’s gimbal (https://www.dji.com). The acquired aerial imagery was tested and compared with the RGB-D on-ground system. The UAV flew autonomously following the programed route by an internal GPS receiver using Litchi APP. The route was set up to take images at an interval of 1 s, creating minimum overlaps of 90% forward and 60% sidewards, at 30 m of flying altitude, and ensuring a necessary overlapping between images for photogrammetry post-processing, mosaicking and Digital Surface Model (DSM) generation.

**Figure 5:**
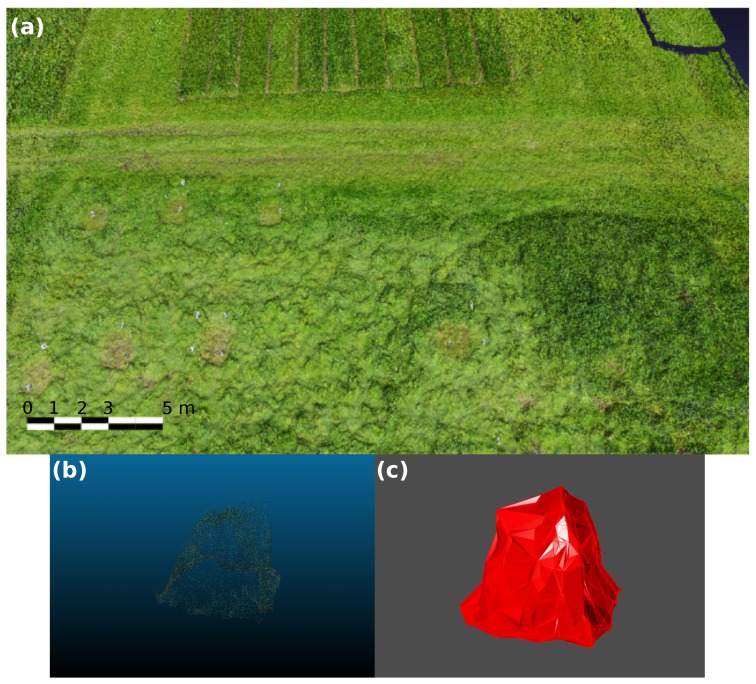
Model constructed by photogrammetry methods (**a**) and processes of point cloud of DSM model (**b**) and solid generation (**c**).

Agisoft PhotoScan Professional Edition (Agisoft LLC, St. Petersburg, Russia) version 1.0.4 was used for 3D model building. This software provides a fully automatic process for image alignment, building field geometry, and orthophoto generation. Quality analysis of all acquired images was done with this software, and images with a value higher that 0.7 were used to reconstruct the DSMs by photogrammetry process. The whole process was fully automatic, except for the manual location of reference points used to correct the model. The model building included several phases: acquisition of very high spatial resolution images with the UAV, and importing them into the software; image alignment; building field geometry by applying close-range photogrammetry methods; dense point cloud generation; application of advanced image analysis to extract the selected geometric features. After that, common points and camera position for each image were located and matched to ensure the refinement of the camera calibration parameters. Then, the software searches for more points in the images to create a dense 3D point cloud, followed by the creation of 3D polygon mesh, from which the final model was generated (Figure 5a).

The DSM and orthomosaics were joined to create a 4-band multi-layer file, i.e., RGB bands and DSM. This file was processed using an OBIA algorithm developed with the eCognition Developer 9 software (Trimble GeoSpatial, Munich, Germany). The software tool for image segmentation and classification applies the multiresolution algorithm Otsu’s of automatic thresholding. 3D features (volume) were calculated by integrating the volume of the individual pixels below the top of the crop as a solid generated object (Figure 5b,c, respectively) [36]. This technique has been successfully applied in UAV images both in agriculture and grassland, as well as urban areas and forestry. A desktop computer equipped with an Intel Core i7-4771@3.5 GHz processor, 16 GB of RAM, and NVIDIA GeForce GTX 660 graphic card was used for image processing and 3D modeling.

After sensor data acquisition, actual height of every sampled plot was determined with the aid of a measuring tape, on the four quadrants plus the center of each plot. Additionally, the compressed sward height was determined using a rising plate meter, which represented the average height at each sampling plot. The compressed height is used by pasture managers as an indicator of the herbage yield, for decision support. Thereafter, all plants inside in the sampling plot were cut at ground level, then oven-dried at 80 degrees Celsius during 48 h, and finally the dry biomass was measured. The calculated ground-truth data was compared with that extracted from 3D models. From the Kinect-based models, plant volume, maximum height, average height and cover area were extracted.

### 2.2. Statistical Analysis

Actual field measurements of plant height and dry biomass were compared with the RGB-D-based and UAV-based 3D models assessments, within each field. Simple linear regression were the tested on all relationships, using the Pearson’s correlation and $R^{2}$ coefficients, with their corresponding standard errors in the evaluation for best fit. Differences between both assessed fields were determined through Anova and subsequent lack-of-fit tests for linear regression models.

## 3. Results and Discussion

### Plant Height, Volume and Biomass

The studied sampling systems differed in data resolution, thus differences were also visible for the estimation capability. Accurate measurement of plant height and volume in pastures is difficult, because single grass plants vary enormously in height, even within areas as small as 0.25 m^2^. Measurements of compressed sward height with a raising plate-meter or of undisturbed sward height with a measuring tape disregard such variation, as well. The former bends down the largest leaves to a ‘common height’ (comparable to an average value) at which all grass plant tips support the plate weight. Similarly, using a measuring tape is based on an ‘average height’, but determined visually. Despite this difficulty, there was a reasonably good agreement the RGB-D-based estimates and the manual height measurements on both fields. These relationships were stronger for the averaged estimates by 1 m^2^ sampling plots, and to a lesser extend for the measurements at the four quadrants. Poor quality of UAV-images resulted due to difficult weather conditions for flying, typicall from the south-wester Norwegian region were this study was carried out. Consequently, UAV-based plant heights could not be reliably estimated, but volume was used instead to evaluate the system. The volume was calculated from the 3D surface, using the alpha shapes (Figure 4). An alpha shape represented the outline surrounding a set of 3D points. Spheres of radius alpha, which did not contain any point inside were generated, and in turn, their surfaces were in contact with more than one point. After connecting those points with the ones of the nearest spheres, the surrounding outline was made, generating the volume. The alpha parameter specified how tight the outline to the points was. Although the height measurement could be done, the exact positioning within the frame in the model was difficult to locate. The height varied significantly as the plot was positioned, resulting in false measurements, consequently, height values were avoided as validation information. The actual plant height (raising plate-meter) averaged for the 20 sampled plots was 49.37 cm while UAV measurements underestimated on average 6.18 cm on the 20 reconstructed models.

**Figure 6:**
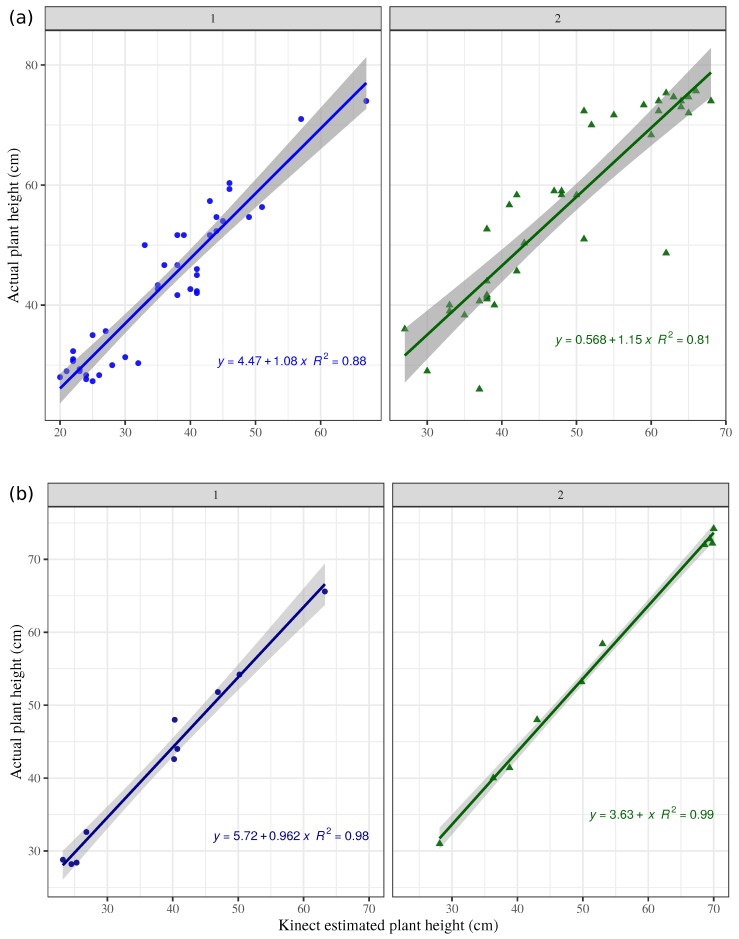
RGB-D estimated grass height compared with field measurements on all four quadrants per sampling plot (**a**), and raising plate-meter height per sampling plot (**b**), on fields 1 and 2. Shadow indicates upper and lower confidence limits.

RGB-D height assessments by quadrant in sampling plots showed a good linear relation between the measured heights at each quadrant, with $R^{2}=0.88$ for field 1 and $R^{2}=0.81$ for field 2 (Figure 6a). This relationship improved greatly when the assessments were averaged per sampling plot $R^{2}=0.88$ and 0.99, respectively for fields 1 and 2 (Figure 6b). Although end-details of grass plant leaves were difficult to reconstruct in the model, the RGB-D-based system showed its powerful capability to estimate accurate height measurements, which is suitable even in small sampling areas, like those in the present study. Field measurements with a raising plate-meter or using a measuring tape also disregard such end-details. Similarly, UAV missions could not reconstruct the end-details. However, good relationship agreements have been found between UAV-estimated heights and ground-truth in other studies [1,37].

UAV-based plant height estimation generally offers two major advantages over the on-ground technologies. UAV can be properly defined as non-destructive monitoring method, and UAVs can cover huge areas in short time. Technically, the use of on-ground measurements should not be considered fully non-destructive when a whole field is to be scanned. Driving field vehicles to carry out the assessments would lead to a high sward biomass destruction, because of the absence of appropriate sampling pathways across the grass field. On the other hand, on-ground monitoring is more time consuming and of a higher operational cost than aerial inspections with UAVs. These type of results was found using multi-temporal crop surface models derived from UAVs and from a terrestrial laser scanner (TLS) [37]. The crop density was well related with the 3D model reconstructions, but differences between the studied methods were evident. Comparable to our results, UAV-derived plant height was generally lower than TLS estimations at all growth stages. However, the coefficient of variation was expected to be higher for the TLS than in those models created from UAV data [37]. Furthermore, Bareth and Schellberg [1] showed the temporal stability of UAV measurements in grassland fields using Structure from Motion and Multi-view Stereopsis techniques, reaching an overall agreement of $R^{2}=0.86$ between rising plate meter heights and model estimations.

**Figure 7:**
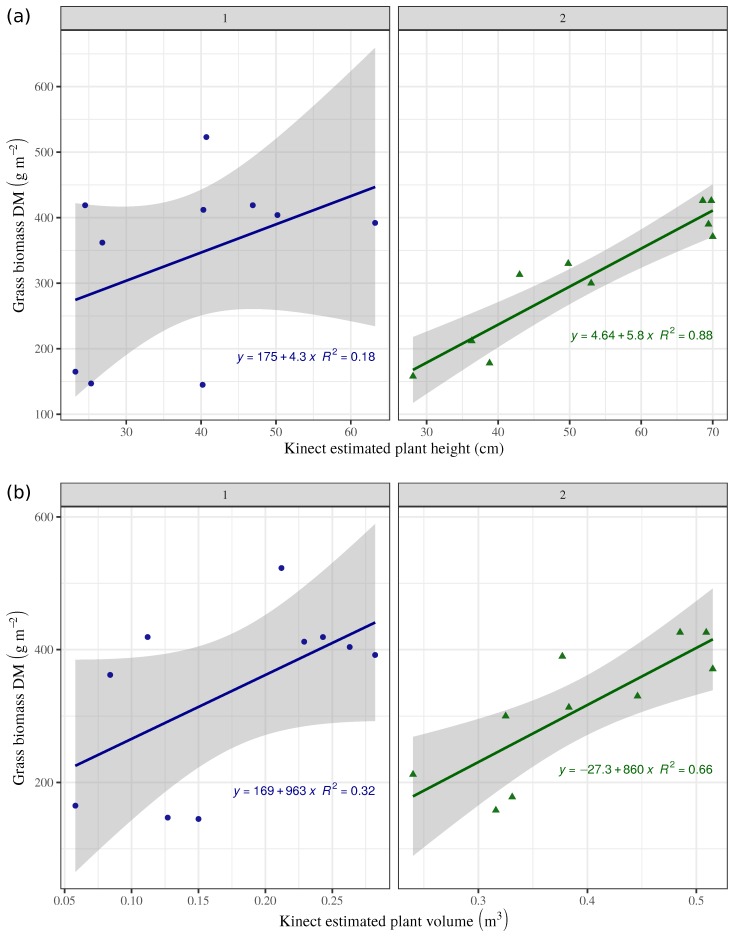
RGB-D estimated grass height (**a**) and volume (**b**) compared with dry biomass per sampling plot, on field 1 and 2. Shadow indicates upper and lower confidence limits.

RGB-D-based grass heights correlated poorly with actual dry biomass on the ryegrass field (Figure 7a, left). This results indicate that plant height is a weak proxy for grass biomass on ryegrass dominant pastures. Conversely, the correlation between RGB-D estimated heights and measured biomass on the timothy dominant field showed a much better fit, with an $R^{2}=0.88$ (Figure 7a, right). These differing results may be explained by the different growth habit of the two species. In the studied fields, timothy built biomass primarily by growing tall, whereas ryegrass built biomass only partly by growing tall, but more by tillering and development of biomass close to ground. However, the models created from the UAV system showed more stability regarding the relationship between dry biomass and plant heights. Equivalently to height measurements, aerial models provide a baseline to avoid the noise caused by some leaves or steams above the average coverage.

In general, the RGB-D sward height estimates were slightly lower than assessments with measuring tape and raising plate-meter heights. UAV systems seem to offer highly reliable assessments, closer to the reality. Nevertheless, capturing fine details on grass plants, such as tips of leaves, would require low flying heights, increasing the amount of images to be acquired and enormous computational power needed for the corresponding analysis. These aspects would therefore, increment the risk of disturbing the estimation results. This effect was more common in UAV flights. The use of on-ground methods could improve the models in some breading programs when high fidelity is demanded, keeping in mind that these type of agronomic applications need fast and higher scanning capacity with non-destructive methods.

**Figure 8:**
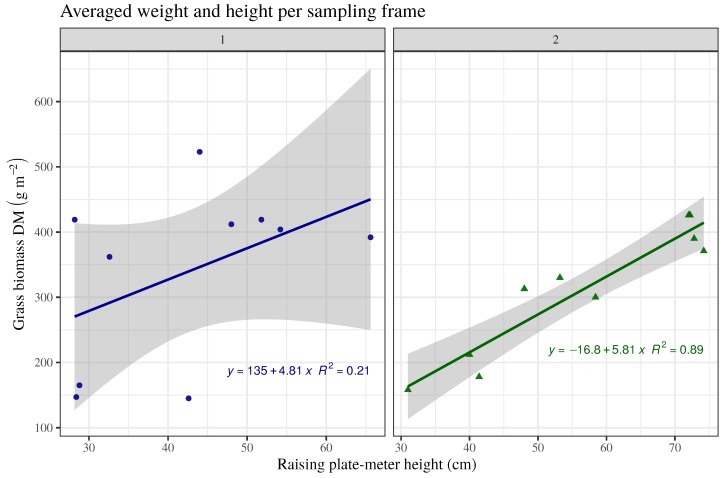
Actual plant height (raising plate-meter) averaged by plot compared with average dry biomass. Shadow indicates upper and lower confidence limits.

RGB-D-based volume estimates showed low and intermediate correlation with the assessed grass biomass in fields 1 and 2, with $R^{2}=0.32$ and 0.66, respectively (Figure 7b). Apparently, the higher content of leaves in ryegrass (Figure 7b, left), contributes more to biomass than to the visible and measurable plant volume to which plant height contributes more than plant density. Conversely, biomass on the timothy dominant pasture (Figure 7b, right), corresponded better with the RGB-D-based volume estimates, as it built yield primarily by growing tall. This same trend was observed comparing the actual measured data of plant height and biomass produced, where this relationship was rather poor for ryegrass (Figure 8, left), while it was good for timothy (Figure 8, right).

A different tendency was observed for aerial models. Volume estimated with the UAV-system on the 20 reconstructed plots had a mean value of 0.39 m^3^ and a standard deviation on 0.17 m^3^ (min = 0.15; max = 0.67). The created models showed an intermediate agreement between the assessed grass biomass and the calculated volume, with an $R^{2}=0.54$. This result shows good capabilities of this method for volume calculation. The developed models showed an irregular shape of the different plots (Figure 5a) and the typical corridors in the experiment. Thus, the accuracy of this method is high and only a few centimeters were underestimated. In the models is also observed that the procedure for 3-D reconstruction was more problematic on areas with a low canopy density. An analogous problem was found in tree reconstruction of orchards, when visible-light images were used. 3-D structure of some of the trees was not properly built, consequently, the mosaicked images showed some blurry areas [28].

**Figure 9:**
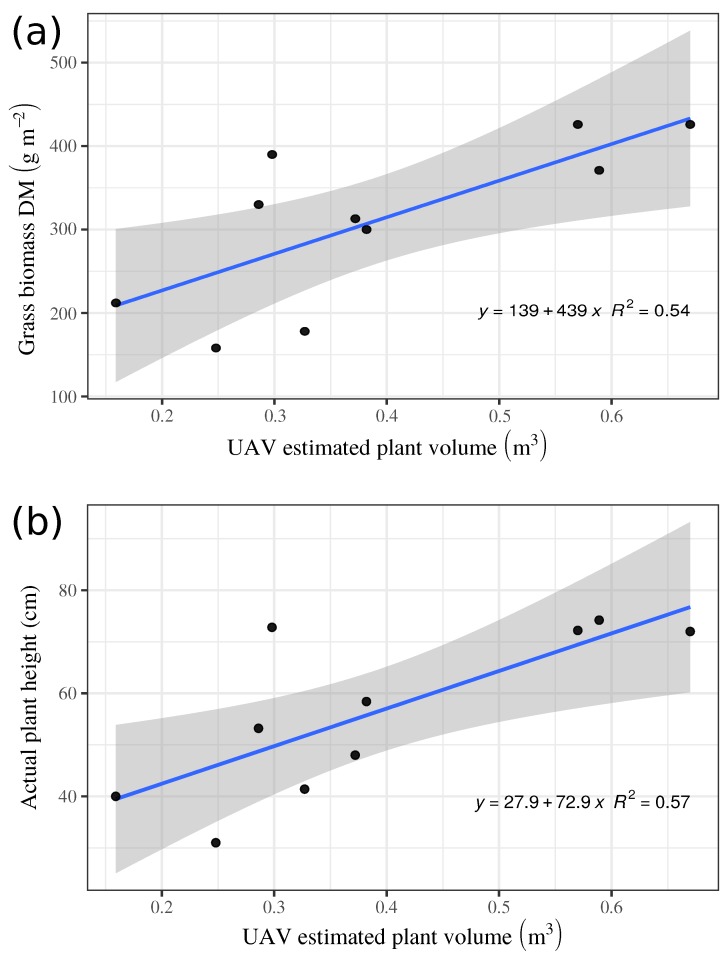
UAV estimated grass volume compared with measured dry biomass (**a**) and with raising plate-meter height (**b**) averaged per sampling plot, on field 1 and 2. Shadow indicates upper and lower confidence limits.

**Figure 10:**
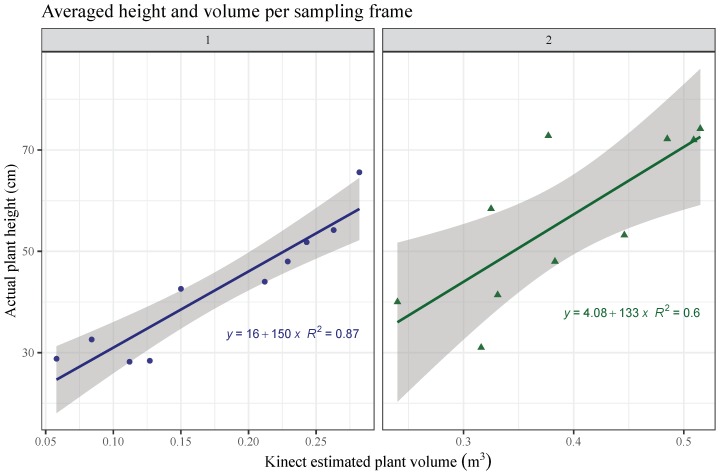
RGB-D estimated grass volume compared with raising plate-meter height averaged per sampling plot, on field 1 and 2. Shadow indicates upper and lower confidence limits.

The estimated grass volume and the rising plate meter height (average height) per plot showed identical values for both, aerial and on-ground methods. Plant volume estimated with UAV system correlated somewhat low with plant dry biomass ($R^{2}=0.54$, Figure 9a). The aerial model showed a correlation between both values of $R^{2}=0.57$ (Figure Figure 9b). RGB-D estimated plant volume for the ryegrass dominant pasture showed a high correlation ($R^{2}=0.87$) with plant height measured and averaged per plot (Figure 10, left), but just an intermediate correlation ($R^{2}=0.6$) for the timothy pasture (Figure 10, right). The timothy pasture showed much more variability in height among individual tillers within the 0.25 m^2^ quadrants (Figure 6), which may explain the low correlation with estimated plant volume for this species. Even though ryegrass plants had a higher number of leaves occupying more volume than timothy per unit area, their leaves bent almost uniformly to a common plant height, which was better measured by the RGB-D system. This fact have been shown in similar studies. A good plant volume estimation using UAV-based image analysis was showed for small weeds [36]. In addition, the combination with multispectral images could improve the results. Estimating above-ground biomass helped monitor crops to predict yield in cereals [29]. The method was proven to be reliable in several scenarios, for instance, relating model biomass estimations to crop nitrogen requirements.

Comparing costs of UAV-based with RGB-D-based systems, considerable differences exist. It has been argued that cost of aerial imaging is lower and can cover bigger areas [38]. The advantage of using UAV-based sampling was notorious in our study, where whole-field coverage could be achieved in less than 12 min. Contrarily, the RGB-D-based system needed considerably more time for all sampling plots. However, the RGB-D-based system in grassland production could be mounted on a tractor and monitoring can be done simultaneously with other agronomic operations, e.g., fertilization or reseeding, thus diminishing the cost.

## 4. Conclusions

The use of UAV-based sampling systems offer a higher operative capability, being also affordable from an economic point of view. This system is more affordable, easier to operate and can cover a larger surface than on-ground systems. Since the resolution for agricultural operations does not need fine identification the end-details of the grass plants (i.e., tips of leaves), the use of aerial platforms could result a better option in grasslands. However, resolution of UAV acquired imagery is affected by other conditions external to the camera sensor, such as sunlight, clouds, wind speed and climate, which also affect the imagery resolution and thus the models for parameter estimations. Conversely, on-ground techniques with RGB-D cameras can produce highly detailed models. Nevertheless, far from higher fidelity models, the results showed more variability than UAV models. Increasing speeds for on-ground platforms would improve the performance of these systems, to monitor more area. On-ground RGB-D data can be effectively analysed with open source software, as it was done in this study, which may compensate and challenge the expenses, compared with aerial sampling. However, this technique can be destructive in pasture scenarios. Although not part of this study, the use of on-ground reconstruction method could be more reliable for row-crops or breeding programs. Particularly, the inclusion of depth information in vegetation models, could contribute to improve the results in breading programs.
